# A Review of *Vaccines for Neglected Pathogens*: *Strategies, Achievements, and Challenges – Focus on Leprosy, Leishmaniasis, Melioidosis, and Tuberculosis* by Myron Christodoulides

**DOI:** 10.4269/ajtmh.24-0807

**Published:** 2025-04-29

**Authors:** Christian T. K.-H. Stadtländer

**Affiliations:** Independent Researcher, Destin, FL 32550 E-mail: ctkstadtlander@msn.com

The term “neglected pathogens” (NPs) is commonly used in association with neglected tropical diseases (NTDs). The World Health Organization (WHO) classifies around twenty NTDs and states: “NTDs are ancient diseases of poverty that impose a devastating human, social, and economic burden on more than 1 billion people worldwide, predominantly in tropical and subtropical areas among the most vulnerable, marginalized populations.”^1^ To end the neglect of these diseases and follow the road map of the “2030 Agenda for Sustainable Development,” global efforts are needed to prevent and control NTDs.^1^^,^^2^ A new book, *Vaccines for Neglected Pathogens: Strategies*, *Achievements*, *and Challenges – Focus on Leprosy*, *Leishmaniasis*, *Melioidosis*, *and Tuberculosis*, edited by Myron Christodoulides, focuses on vaccine development for the following NPs: *Mycobacterium tuberculosis*, *M. leprae*, *M. ulcerans*,* Leishmania* spp., and *Burkholderia pseudomallei*.^3^ All these pathogens have in common an intracellular phase in human host cells. The book highlights the activities of a group of international researchers of the Vaccine Development for Complex Intracellular Neglected Pathogens (VALIDATE) network. The network aims at accelerating vaccine development for NTDs and facilitating career advancement of network investigators, especially of early career researchers.^4^ The editor divided the book into four parts with a total of 15 chapters. The first two chapters serve as an introduction.

In Chapter 1, Christodoulides reviews the WHO list of NTDs and their causative agents, which include viruses, bacteria, parasites, fungi, and helminths. He then compares the funding for research and development into NTDs, detailing expenditures for basic research, drugs, vaccines, diagnostics, and vector control products. Chapter 2 describes the creation and expansion of the VALIDATE network. Vermaak et al. mention that the network was established in 2017 and now connects members from over 70 countries. They review VALIDATE’s focus on NPs and describe the network’s funding sources, fellowship recipients, and training grants.

Part I (Chapters 3–5) deals with mycobacterial diseases, including leprosy and Buruli ulcer (BU). Slater points out in Chapter 3 that leprosy affects over 200,000 people each year and is endemic in some regions of the Americas, southeast Asia, and Africa. An infection with *M. leprae* can cause severe nerve and skin damage, leading to permanent deformity and disability. In January of 2024, a global appeal was made by the WHO to “end stigma and discrimination against persons affected by leprosy.”^5^ Chapter 4 discusses treatment options for leprosy. Wang describes the use of multi-drug therapy and immunization with the Bacille Calmette-Guérin (BCG) vaccine. Although the BCG vaccine was originally developed to treat tuberculosis, it is effective against leprosy. Wang discusses recombinant BCG and protein subunit vaccines as well as vaccine-drug combinations for the treatment of leprosy. In the fifth chapter, Boakye-Appiah et al. review the epidemiology, clinical presentation, and treatment options for BU, which is a necrotizing and disabling skin disease caused by the mycolactone toxin of *M. ulcerans*. The authors explain current treatment regimens with antibiotics (e.g., clarithromycin) and the prospect for a BU vaccine. Although there is currently no effective vaccine to provide long-term protection against BU (the BCG vaccine appears to provide some protective immunity), there are several human trials underway with novel vaccine candidates.

Part II (Chapters 6–10) focuses on prophylactic and therapeutic interventions to control tuberculosis. Alvarez et al. describe in Chapter 6 the multiple immunological interactions that take place during an infection with *M. tuberculosis*. They explore the role of innate immunity, T-cell activation, and humoral responses to this pathogen. Furthermore, they explain the value of animal models and vaccine clinical trials, and the usefulness of controlled human infection models (CHIMs). The latter includes a purified protein derivative (PPD) CHIM and a BCG CHIM. In the seventh chapter, Li and Li assess established and new animal models for the study of tuberculosis. They state: “Each animal model cannot mimic completely the symptoms of human tuberculosis, so in practical application, the characteristics of different animals are often complementary to each other to fulfill research aims.” Chapter 8 is titled “BCG: Past, Present, and Future.” Morrison and McShane reiterate some information discussed in preceding chapters but then focus on BCG vaccine safety and adverse effects. Moreover, they discuss existing and alternative routes of administration of the BCG vaccine as well as the potential use of the vaccine for the treatment of viral and non-communicable diseases. Chapter 9 is about the manufacture of the BCG vaccine. Walker and Bacon call attention to the fact that the production of this vaccine has not changed much over the years. They believe that producing the vaccine in a bioreactor could provide many benefits, including faster and more reproducible production that would deliver the vaccine at a higher yield and in a form that would be easier to characterize. The authors provide valuable information about regulatory requirements for novel BCG vaccines. Whitlow et al. continue the discussion of the BCG vaccine in Chapter 10 by focusing on the identification and characterization of immunological markers for *M. tuberculosis*. They review several types of vaccines, including live attenuated, inactivated, subunit, and recombinant vaccines to control *M. tuberculosis* infection.

Part III (Chapters 11–14) is about leishmaniasis. In Chapter 11, Osuolale reviews information on *Leishmania* (a protozoan parasite), transmission routes, and distribution and clinical manifestations of tegumentary leishmaniasis (TL) and visceral leishmaniasis (VL). He explains treatment options, including the use of anti-leishmanial drugs (e.g., amphotericin B, miltefosine, and paromomycin) and vaccine development approaches for TL and VL. Several generations of vaccines against *Leishmania* species have been developed, but only a few have reached the clinical trial phase. Chapter 12 is a re-visit of the usefulness of CHIMs, discussed here for the testing of leishmaniasis vaccines. Kaye et al. argue that CHIMs “uniquely offer delivery of important insights into the pathogenesis of disease (including characterisation of the incubation period), the identification of early correlates of protection or disease progression and a clearer understanding of the relationship between pathogen load, immunity, and transmission.” Chapter 13 is about canine leishmaniasis (CanL). Coelho and Christodoulides explain that CanL is a zoonotic vector-borne disease that is transmitted by the bite of an infected sandfly. The diagnosis, treatment, and immune responses to CanL as well as licensed and experimental CanL vaccines are reviewed. In the fourteenth chapter, Clímaco et al. summarize information about human leishmaniasis and speculate whether aspects of the development of COVID-19 vaccines can facilitate development of VL and TL vaccines.

Part IV (Chapter 15) is about vaccine development against melioidosis. Galeas-Pena and Morici describe the biology and virulence factors of *B. pseudomallei*, the causative agent of melioidosis. This disease can be asymptomatic, mildly symptomatic, or life-threatening when developing into pneumonia or sepsis. Several vaccine development approaches for melioidosis are discussed, including live-attenuated and inactivated whole-cell and multi-valent or conjugate subunit vaccines. The authors mention that several promising vaccine candidates are in the pipeline to control melioidosis, including those containing adjuvants.

This multi-authored book contains a wealth of information about NPs and associated NTDs. It provides readers with a detailed review about the history and geographical distribution of tuberculosis, leprosy, leishmaniosis, and melioidosis. The authors describe in a remarkable way the biology of *Mycobacteria*, *Leishmania*, and *Burkholderia* species, and provide invaluable information about the immunological responses to infection with these pathogens. Furthermore, there is plenty of information provided about pre-clinical and clinical trials, which allows readers to understand the scope of multi-disciplinary basic and applied vaccine research.

One of the key take-aways from reading this book is being reminded how widely the BCG vaccine was/is used for controlling tuberculosis (especially for protecting children from severe forms of the disease) and for cross-protecting against other mycobacterial diseases (leprosy and BU). Furthermore, the prospect of using BCG vaccine preparations to enhance protection against non-communicable diseases (e.g., cancer, allergies, autoimmune, and inflammatory diseases) as well as viral diseases (e.g., corona- and filoviruses) shows the versatility of this vaccine.^6^^–^^8^ Thus, this century-old BCG vaccine can offer clues about specific and non-specific immunological responses in a host and can lead to the design of future vaccines.

I consider a limitation of this book to be insufficient discussion of the ethical requirements for conducting vaccine research and development. The authors mention some of the ethical concerns of animal and human experimentation, especially for the use of CHIMs, but do not discuss how they can be addressed. I believe a chapter about the ethics of pre-clinical and clinical trials should have been added to the book, including a discussion about the concerns of accelerated trials during the 2019 coronavirus pandemic. Readers can access information about this topic elsewhere.^9^^,^^10^ Other limitations include the lack of a glossary or an index for keyword searches.

Despite some shortcomings, I consider this book an important contribution to the literature on NTDs. It can be used as a valuable resource for researchers in academia and industry, clinical investigators, and public health professionals. In addition, it is suitable as a learning tool for graduate, medical, and veterinary students enrolled in courses such as microbiology, immunology, infectious diseases, and international health. Furthermore, funders for NTD research and policy makers for tropical diseases should read this book.
